# Role of the blood–brain barrier in the evolution of feeding and cognition

**DOI:** 10.1111/j.1749-6632.2012.06568.x

**Published:** 2012-05-21

**Authors:** William A Banks

**Affiliations:** GRECC, Veterans Affairs Puget Sound Health Care System and Division of Gerontology and Geriatric Medicine, Department of Medicine, University of Washington School of MedicineSeattle, Washington

**Keywords:** Blood–brain barrier, leptin, feeding, cognition, obesity, central nervous system, evolution

## Abstract

The blood–brain barrier (BBB) regulates the blood-to-brain passage of gastrointestinal hormones, thus informing the brain about feeding and nutritional status. Disruption of this communication results in dysregulation of feeding and body weight control. Leptin, which crosses the BBB to inform the CNS about adiposity, provides an example. Impaired leptin transport, especially coupled with central resistance, results in obesity. Various substances/conditions regulate leptin BBB transport. For example, triglycerides inhibit leptin transport. This may represent an evolutionary adaptation in that hypertriglyceridemia occurs during starvation. Inhibition of leptin, an anorectic, during starvation could have survival advantages. The large number of other substances that influence feeding is explained by the complexity of feeding. This complexity includes cognitive aspects; animals in the wild are faced with cost/benefit analyses to feed in the safest, most economical way. This cognitive aspect partially explains why so many feeding substances affect neurogenesis, neuroprotection, and cognition. The relation between triglycerides and cognition may be partially mediated through triglyceride's ability to regulate the BBB transport of cognitively active gastrointestinal hormones such as leptin, insulin, and ghrelin.

## Early experiments in obesity

In 1950, Ingalls *et al.* reported in *Journal of Heredity* a mouse that had a recessive trait for obesity and dubbed it the *ob/ob* mouse.^1^ Differences between affected and unaffected littermates were obvious by 3 weeks of age, but by 10 months of age, unaffected littermates weighed about 29 g, whereas affected mice weighed about 90 g, a threefold difference. Parabiosis experiments 20 years later showed that obesity in these mice was caused by the absence of a circulating factor.^2^ Some 20 years after the parabiosis experiments, Freidman *et al*. identified that missing factor as leptin.^3^,^4^

Unlike the *ob/ob* mouse, almost all obese humans have high, not low, levels of leptin.^5^ Such conditions in which the regulatory hormone is high in the face of a deficient response are termed in endocrinology *resistance syndromes*.^6^ Thus, obesity in humans is dominated by a resistance to, not a deficiency of, leptin. In classic resistance syndromes such as pseudohypoparathyroidism, the first resistance syndrome to be described, resistance arose because of defects either in the receptor or in the receptor's intracellular machinery.

The *db/db* mouse lacks leptin receptors and is obese and so represents a classic case of receptor/postreceptor resistance.^7^ However, the negative feedback loop formed between leptin and body fat has two steps not found in peripheral tissues. The first of these is at the blood–brain barrier (BBB). As leptin is a large molecule, a protein of 16 kDa, its transfer from blood into the brain is aided by a transport system. The second are the downstream neural circuitries: in other words, leptin acts on other cells and modulators such as melanocortin^8^ to mediate its effects on body weight. Defects in either the ability of the BBB to transport leptin or in the response of the downstream neural circuitries to respond to leptin leads to the resistance picture of high serum leptin levels in the face of obesity.

## Transport of leptin across the BBB in obesity

Early work showed that acquired defects in the BBB transport of leptin played a role in obesity. In 1997, two publications showed that animals with diet-induced obesity went though a phase when they no longer responded to leptin given peripherally but still responded to leptin given directly into the brain.^9^,^10^ This was evidence not only that leptin was crossing the BBB in ineffective amounts, but also there existed a phase in which resistance at the BBB (termed *peripheral resistance*) was functionally dominant to resistance at the receptor/postreceptor level (termed *central resistance*). Subsequent studies from several laboratories have shown pharmacokinetic impairment in the blood-to-brain transport in several models of obesity including obesity of maturity, diet-induced obesity, the obesity prone rat, and the Koletsky rat; the latter two being genetic models in which leptin receptors in brain are deficient or absent.^11^–^16^ The obesity prone rat of Levin are born with defects in CNS leptin receptors but normal transport of leptin across the BBB.^11^ As they develop obesity, they acquire impairments in leptin transport. As these studies would predict, pharmacologic induction of peripheral resistance with an agent that prevents endogenous leptin from crossing the BBB stimulates feeding and body weight gain.^17^ These effects begin immediately with the onset of treatment and result in a body weight gain that is essentially all attributable to an increase in adiposity.

Calculations in the CD-1 mouse and experiments in sheep have suggested that peripheral resistance in diet-induced obesity accounts for all or the majority of leptin resistance.^18^,^19^ In other models and in humans, results show that leptin resistance clearly has both peripheral and central components. The finding in humans that leptin levels in cerebrospinal fluid are higher in obesity than in thin persons clearly demonstrates resistance at the receptor level.^20^,^21^ Mathematical modeling based on classic assumptions of receptor resistance suggests that at low to moderate levels of obesity, peripheral resistance dominates.^18^ The modeling also suggests that as obesity increases, resistance at both sites progressively worsens.

**Figure 1:**
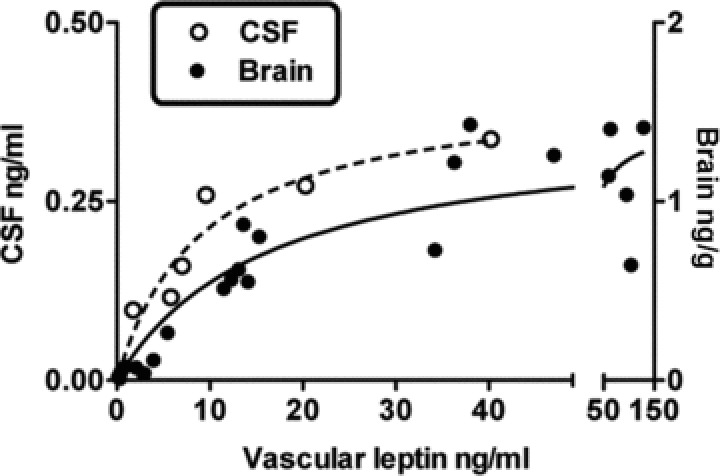
Relation between vascular and CNS levels of leptin. CSF versus blood levels of leptin (open circles, dotted line) are derived from several studies of humans in the literature.^20^–^22^ Brain versus vascular levels of leptin (solid circle, solid line) are derived from a brain perfusion study.^23^ Either approach shows a hyperparabolic relation between CNS and vascular levels of leptin that is explained by the saturable nature of the blood-to-brain transport of leptin across the BBB. With either approach, the curve shows significant saturation at relatively low vascular levels of leptin.

The mechanisms of leptin resistance at the BBB are multiple. First, the saturable nature of leptin transport means that there is a serum level at which leptin transport into brain is at its maximum. CSF versus serum data from human studies^20^–^22^ and brain versus serum data from perfusion studies in mice^23^ independently show that the effects of saturation are seen at serum levels of 5–10 ng/mL (Fig. 1). In other words, the transport of leptin across the BBB is most efficient when serum levels are low.

Other mechanisms of resistance relate to the regulated nature of leptin. Several factors modulate the transport rate of leptin across the BBB (Table 1). In the case of lipopolysaccharide treatment, the effect is indirect in that it is mediated by an elevation in leptin levels.^24^–^26^ In the case of triglycerides, the effect appears to be direct in that triglycerides inhibit leptin transport across *in vitro* models of the BBB.^27^ Other factors give clues to the physiological role that leptin plays in energy homeostasis. For example, short-term fasting restores the leptin transport deficit in obese mice, whereas long-term fasting (starvation) inhibits transport.^14^,^28^ These findings are thought to be driven by triglyceride levels that decrease in fasting but increase in starvation.

**Table 1:** Regulators of leptin transport across the BBB

State or substance	Effect on transport	Reference
Obesity	Decreased	12, 15, 16
Starvation	Increased	28
Fasting	Decreased	27
Epinephrine	Increased	67
Insulin	Increased	68
Glucose	Increased	68
Triglycerides	Decreased	27
Ovarectomy	Decreased	69
Alcohol administration	Increased	70
Lack of leptin receptor	Decreases	11, 15
Defects in leptin receptor	Decreased	11
Diurnal rhythm	Variable	71

Therefore, the relation between body weight status and leptin transport rates across the BBB have both chronic and acute influences. At a stable steady state, obesity and serum leptin levels relate to BBB transport rates and brain levels of leptin. But in starvation, even before significant weight loss has occurred, serum leptin levels the become uncoupled to body weight.^29^ With the elevation in triglycerides that occurs with extended periods of fasting, serum levels and transport rates become uncoupled.^30^ Although a high level of serum leptin is a poor anorectic, a low brain level of leptin is a powerful orexigen. Hence, the leptin/BBB/brain axis seems geared to promote feeding, especially during times of low food intake, but not to inhibit feeding during times of plenty.

## Leptin and energy balance: an evolutionary perspective

Early work that attempted to frame leptin actions as an adipostat were frustrating.^31^,^32^ The perspective that leptin is most relevant when body fat reserves are low is a more versatile concept. In starving animals or animals with very low serum leptin levels, calorically expensive activities such as reproduction and support of the immune system are curtailed, and low doses of leptin will allow these activities to resume.^33^–^37^ This suggests a view that leptin acts as an adipometer; that is, it informs the brain of the magnitude of caloric reserves. The brain then determines whether behavior and calories are best spent on seeking and ingesting food or whether caloric reserves are to be spent in other activities.

Various lines of evidence support the low-level permissive model of leptin action. As discussed earlier, it is at low serum levels of leptin that the BBB transporter for leptin works most efficiently. Most animals living in the wild have only 3–7% of their body weight as fat mass and serum leptin levels of 1–2 ng/mL.^38^–^42^ Likewise, modern hunter gatherers as illustrated by the !Kung San have body mass indices of 19.1 for women and 19.4 for men.^43^ These values are much lower than those considered optimal, normal, or ideal in Western societies, but are in the region where the BBB leptin transporter most efficiently conveys information to the brain.

There has been tremendous evolutionary pressure to safeguard against starvation, but probably little pressure to deal with problems of obesity.^44^ As the Paleolithic diet was low in fat, simple carbohydrates, and salt, mechanisms from behavior to metabolic pathways adapted to obtain and maximize use of these important nutritional resources. An adipometer would have been needed to constantly inform the brain of whether calories were sufficient to devote to activities other than seeking food or whether caloric reserves were approaching the starvation range. Even though most modern hunter-gatherers live in tropical areas, they still face seasonal variations in food supply and, as a result, variations in body weight and fat mass.^45^

With this view, inhibition of transport by starvation and by triglycerides is explicable in metabolic and evolutionary terms. With starvation, triglycerides stored in adipose tissue are hydrolyzed to their free fatty acids.^46^ Some of these free fatty acids are used to synthesize new triglycerides so that blood levels of both free fatty acids and triglycerides are elevated in starvation. A theoretical scenario can then be constructed from the premise that triglycerides evolved as a signal of stravation to the brain: a drop in caloric intake is sensed; adipose tissue responds by decreasing its production of leptin; thus serum leptin and brain levels of the leptin decrease. As starvation continues, serum triglycerides increase that impede leptin transport across the BBB; hence, brain levels of leptin decrease further. Decreasing leptin levels in the brain remove the permissive effect that allows calories to be spent on nonfeeding activities and redirects caloric use towards food-seeking activities.

## Integration with other feeding signals

Many peptides and regulatory hormones have influences on feeding. Indeed, it may seem odd at first that so many factors control, regulate, or influence an act that at first seems as simple as eating. But ingestion, digestion, excretion, and related activities are, in reality, extremely complex acts. Digestion requires complex coordination of enzymatic, secretory, hormonal, neuronal, and muscular events that are under voluntary and involuntary controls. Many of the monoamines, peptides, and regulatory proteins known to affect feeding and digestion have specific functions that must be closely coordinated with other events.

Complexity of feeding also involves higher order cognitive functions. Berthoud has summarized neuronal circuitries that weld together ingestive, cognitive, and emotional aspects of feeding.^47^ In Western societies, the decisions of when, where, and what to eat are not usually considered major intellectual challenges. But for animals living in the wild, one of the most complex cognitive-related behaviors in which they engage is the decision related to feeding. During periods when caloric use is high or when food resources are low, reproductive success will improve from even a primitive cost/benefit analysis of whether more calories are likely to be gained than expended in the search and acquisition of food. Primates, reptiles, and birds all illustrate situations in which cost/benefit analysis would be helpful in maximizing caloric acquisition/expenditure. Primates have been found to take recent trends in temperature and solar radiation into account in deciding whether to revisit trees for newly ripened fruit.^48^ Iguanas balance acquisition of food located in cold areas against the disadvantage of leaving a warmer area with less palatable food.^49^ In storing food for future use, mountain chickadees alter their caching strategies when potential pilferers are in the area.^50^ This requires the chickadees to not only assess sites for security, but also evaluate the motives of other chickadees in the area. Studies with New Zealand robins illustrate how a degree of numerical competency can covey significant advantages in food storage, pilfering, and retrieval.^51^

Sickness behavior represents an especially good example of an adaption that balances feeding and caloric demand.^52^,^53^ When animals are ill, short-term conservation of energy by reducing energy expending activities, including feeding behaviors in favor of rest can be beneficial. Calories can be used instead to increase body temperature and otherwise support immune activities.

Cost/benefit analysis is also productive regarding the probabilities of how safe it is to search for food. Whether a top predator or an occupier of a position low on the food chain, seeking and acquiring food can be a dangerous activity. Social rules about acquiring, sharing, or trading food exists in many animal societies and decisions on how to follow those rules or even when to violate them requires cognitive abilities.

Thus, feeding requires complex and contextual decisions. This may explain why so many of the feeding hormones have effects on learning, memory, attention, and other aspects of cognition. MSH, ghrelin, insulin, leptin, and glucagon-like peptide-1 are examples of gastrointestinal hormones with effects on cognition.^54^–^62^ As with feeding, these hormones cross the BBB to exert their CNS effects. It is interesting that triglyceride levels not only affect transport of many of the feeding hormones across the BBB but also correlate with cognition.^63^–^66^

## Conclusions

The BBB plays important roles in the regulation of the blood-to-brain transport of peptides and proteins involved in feeding. Leptin, insulin, and ghrelin are examples of hormones that are transported from blood-to-brain by saturable transport systems. Impaired transport of leptin across the BBB results in obesity and also occurs during starvation. Various mechanisms underlie transport defects of leptin. Triglycerides impair leptin transport and may be adaptive, having evolved to inhibit the CNS effects of leptin during starvation. Many gastrointestinal hormones have effects on cognition. Such effects may again have arisen from evolutionary pressures resulting from the complexity of feeding, especially those aspects that involve social and cost/benefit ratio decisions. These cognitive effects, like the feeding effects, of many of the gastrointestinal hormones are dependent on their abilities to cross the BBB.
